# Surgical Cases in the Hospital at Milan

**Published:** 1860-01

**Authors:** 


### SELECTIONS.

*Surgical Cases in the Hospital at Milan.*

[Prof. Paul F. Eve, of Nashville, Tenn., who has recently returned from a tour in Europe, visited, while there, the places which have been made famous by the events of the late Italian war. The hospital at Milan, in which many of the wounded were received, with some notice of the more interesting cases observed, is thus spoken of in Dr. E.'s correspondence with the *Nashville Journal of Medicine and Surgery.*]

We were fortunate in procuring the services of a sub-officer, who described the particulars of the battle fought at Magenta on the 4th of June. To some soldiers who returned to the railroad station while we were there, he asked, "have you seen any feet to-day?" Upon inquiry what was meant, he replied that even up to the present time, fifty-two days since the attack, owing to the heat and the slight covering of earth, corpses were occasionally exposed.

We arrived at Milan, about twelve miles beyond Magenta, at 2, P. M. Here 17,000 wounded have been received, from Solferino alone. At St. Ambrose there were 1,700 at one time, and 700 still remain. Baron Larrey, son of the old Baron under Napoleon I., to whom Dr. Dunglison kindly gave me a letter, and whom I knew in Paris as a student twenty-five years ago, told us there were twenty-one hospitals in Milan. He gave orders for our reception at every one, but, ot course, as we could not see all, we choose the three largest and most interesting.

In the two days spent here we noted the following cases: At St. Ambrose, built in the sixteenth century, the one of greatest interest was that of a soldier shot twice at Milegnano, more than forty days ago. One ball fractured the third rib, perforating the right lung and traversing the thorax. A second one fractured the surgical neck of the humerus on the same side. No union having taken place, it was determined to exsect the head of the bone. With a double edged knife the track of the bone was followed, and by a crescentic incision from above downward, and from within outward, a large flap was made of the deltoid muscle and integuments. The head of the bone was then taken out of the glenoid cavity, and the bone below the fracture sawed off. The case was doing well, and the surgeon in charge was quite proud of it.

At the great civil hospital, with three thousand beds, we noticed four cases of compound fracture of the thigh, under Surgeon Michili. One had a ball through the upper third of the femur, which was badly shattered. The patient was brought in from Magenta, and this is now the fifty-sixth day since he was wounded. To-day the opening made by the entrance of the ball was enlarged, and gave exit to a large quantity of pus. The limb is in splints. No union of bone. Many spiculæ have been removed. His recovery is doubtful. 2d case.—The ball also fractured the upper third of the femur, and has not yet been found. Incisions have been made, and there is now free suppuration. Result of case also doubtful. The patient complains most of his heel, where there is a large ulceration which has bled occasionally. Was wounded at Magenta. 3d case.—Wounded by a ball through the lower third of the thigh, involving the knee-joint. Has diarrhœa, with free suppuration about the injured parts. Have little expectation of his recovery. 4th case.—The ball traversed the upper part of the left thigh, at Magenta. Is doing well, and there is little doubt of his recovery.

Saw also here a patient coming from Solferino, having a ball to strike the olecranon, and pass out at the inner side of the limb, opening the joint. Doing well, with prospects of anchylosis. A ball traversed one side of the spine without injuring it, and has been lost on the other side. A ball struck the spine of the tibia in another case, and was divided by it, each portion coming out in the calf of the leg. A soldier received a ball at Solferino, to the left of the median line of the upper lip on the left side; it came out in the parotid region. With the exception of a salivary fistula, he is doing well. Another at Milegnano had a ball to traverse the left pleural cavity and come out at the inferior angle of the shoulder blade. He had no hæmoptysis, but there is some suppurative action going on near the wound of exit. This was freely laid open to-day. Another is here with a wound made by a ball passing under the outer third of the right clavicle. The right upper extremity of this side is partially paralyzed, with irregular nervous paroxysms, for which nothing as yet has afforded entire relief. At Solferino, a soldier was struck upon the right zygomatic arch, the globe of the right eye ruptured, and the nose at its base perforated, the ball escaping just under the left eye. Is doing well. Found here also two cases of fractured jaws. The one in the inferior was made by a ball striking the middle of the inferior lip, fracturing the bone, and making its exit near the mastoid process of the right side. This occurred at Magenta. After removing spiculæ of bone, there has resulted good union of the inferior maxillary. In the second case the ball passed through the entire upper jaw, from side to side, including the hard palate. The large opening between the nose and mouth is now closed by a gold plate and artificial teeth. For a ball passing through the ham and injuring an artery, with subsequent formation of an aneurism, the femoral artery was tied at the usual point of selection, on the 20th of July. Apparently doing well. Observed here, too, a case of tetanus. The patient was shot at Solferino, by which the tibia was fractured. This was on the 24th of June. The attack of lockjaw commenced fifteen days ago, and the surgeon in charge ascribes the relief to *muriate of baryta*, given, I think he said, in 6 grains to 20 or 30, three times a day. By this remedy he told me he had cured four cases. The tetanus is pretty much confined to the side wounded. He has, moreover, violent contractions of the leg upon the thigh, and then chloroform is inhaled. The patient is able to take nourishment freely. The last in this hospital which I recorded the notice of, was that of a Captain, with a thigh greatly shattered by a ball at Milegnano, on the 8th or 12th of June. The surgeons proposed at once to amputate the limb by candle light, to which he objected, when he was brought to Milan, and seventeen hours after being wounded the operation was performed in the middle third of the femur. He is now nearly ready to return to la belle France. These four last cases were under the care of medico-chirurgo Gustavo Tassani, of Milan.

At the Hospital of St. Prassede I collected the following facts: A patient wounded at Magenta, had a severe hemorrhage from apparently one of the articular arteries about the knee, 31 days after being wounded. There is now great infiltration in this extremity. I much apprehend here loss of limb or life. The ball passed through the knee-joint. A ball entered left commissure of mouth, fractured the jaw near the angle of the same side, and passing out, went through the neck above the clavicle, and out near the scapulæ. Patient is doing well. At Solferino the elbow joint of a soldier was traversed by a ball, and now, notwithstanding every attention, it has been agreed that amputation must be performed. In another, the same ball passed through both calves of the legs, making four wounds without fracture. The first struck has healed soonest, but in the last one wounded, the union has been interrupted by foreign substance in it, for yesterday a piece of clothing was removed from it. A wrist-joint, perforated through and through, anteroposterior, has been saved, the patient being now nearly well. It is true the bones of the carpus may yet inflame and ulcerate. —*Boston Medical and Surgical Journal.*

